# Establishment of a conditional transgenic mouse model expressing human uncoupling protein 2 in vascular smooth muscle cells

**DOI:** 10.3892/etm.2012.620

**Published:** 2012-06-25

**Authors:** SHUANGTAO MA, DE LI, DACHUN YANG, YAN TAN, BING TANG, FEIPENG JIN, SIHUA JIANG, XIUCHUAN LI, YONGJIAN YANG

**Affiliations:** Department of Cardiology, General Hospital of PLA Chengdu Military Area Command, Chengdu, Sichuan 610083, P.R. China

**Keywords:** uncoupling protein 2, vascular smooth muscle cell, transgenic mouse

## Abstract

Increased oxidative stress is involved in the development of vascular dysfunction and remodeling. Uncoupling protein 2 (UCP2) regulates the production of reactive oxygen species in vascular smooth muscle cells (SMCs). To promote the study of the role of UCP2 in vascular diseases, a transgenic mouse model expressing human UCP2 (hUCP2) in vascular SMCs was established. We constructed a plasmid carrying the 2.3 kb rabbit smooth muscle myosin heavy chain promoter and the hUCP2 gene. We used this plasmid to produce transgenic mice by pro-nuclear microinjection. Six offspring were identified as founder mice that were used to establish a transgenic mouse lineage. The transgenic mice showed a significant increase in hUCP mRNA expression in the aorta. Moreover, hUCP2 overexpression inhibited the production of superoxide and increased the bioavailability of nitric oxide (NO). In this study, we established a hUCP2 transgenic mouse model, which will enable further studies on the role of UCP2 in vascular dysfunction and remodeling.

## Introduction

It has been reported that reactive oxygen species (ROS) are involved in several vascular diseases, including endothelial dysfunction and atherosclerosis (1). Uncoupling protein 2 (UCP2) belongs to the mitochondrial anion carrier family and is expressed in several types of cells, including vascular smooth muscle cells (SMCs) (2). UCP2 is known to be involved in the uncoupling of the proton electrochemical gradient, resulting in the reduced production of ROS. A previous study demonstrated that LDLR^−/−^ mice with UCP2-ablated macrophages had significantly larger aortic atherosclerotic lesions than the controls despite lower cholesterol levels, indicating a protective role for UCP2 in atherosclerosis (3). Lombard reported that UCP2 knockout enhanced the high salt diet-induced increase in superoxide production and decrease in nitric oxide (NO) bioavailability, eliciting a consequent elevation of blood pressure and markedly enhancing the impairment of vasodilation, suggesting that UCP2 plays an important role in the development of salt-related hypertension and vascular dysfunction (4). These studies strongly support the theory that UCP2 is closely related to the development of vascular diseases.

Although the UCP2 is highly conserved between mouse and human, there are species differences over the protein sequence. Other researchers have created transgenic mice that overexpressed UCP2 in hepatocytes (5) and hypocretin neurons (6) for the study of obesity. However, the effect of the overexpression of human UCP2 (hUCP2) specifically in vascular SMCs has not been addressed. In the present study, to facilitate the functional study of hUCP2, we report the establishment of transgenic mice carrying the hUCP2 gene in vascular SMCs. This model provides a tool that may be used to define the role of SMC-derived hUCP2 in the development of vascular disease.

## Materials and methods

### Animal care

Handling of animals and all experimental procedures were approved by the Institutional Animal Care and Use Committee of the General Hospital of PLA Chengdu Military Area Command. Transgenic founder mice were generated on an FVB background. The cDNA from hUCP2 under the control of the rabbit smooth muscle myosin heavy chain promoter was cloned into the pRP.Des2d vector (Cyagen Biosciences Inc., Guangzhou, China) and verified by sequencing. The linearized pRP.Ex2d-SMHC>UCP2 was purified from agarose gel using a QIAquick Gel Extraction kit (Qiagen, Chatsworth, CA, USA), adjusted to a final concentration of 1 mg/ml in Tris-EDTA (TE) buffer and used as a DNA solution for microinjection. The female FVB mice were hormonally superovulated and mated with male FVB mice. Next morning the fertilized one-cell eggs were collected from the oviducts. The eggs were microinjected with the DNA solution under a microscope. The injected fertilized eggs were transplanted into the oviducts of pseudo-pregnant FVB mice. Transgenic mice were identified by PCR with the forward and reverse primers 5′-GGAGATACCAAAGCACCGTCAA and 5′-CATAGGTCACCAGCTCAGCACA, respectively. The internal control was identified by PCR with the forward and reverse primers 5′-TCT TAG CTC TGC TCT CCG GT and 5′-CAC TGG CTG AGG AAG GAG AC, respectively.

### Gene expression analysis

The RNA from the aorta was prepared using the RNeasy mini kit (Qiagen) and reverse transcription and PCR were performed using a QuantiTect SYBR Green one-step RT-PCR kit (Qiagen) according to the manufacturer’s specifications. A standard curve was prepared for each assay by the serial dilution of cDNA synthesized from pooled RNA. The samples and standards were analyzed in triplicate (7).

### Dihydroethidium assay

To assess the levels of superoxide production, the fresh frozen aortae were cut into 20-μm thick sections. Following incubation in Krebs solution for 30 min, the samples were incubated in the dark with dihydroethidium (DHE; Sigma-Aldrich, St. Louis, MO, USA) diluted in Krebs solution (40 μmol/l) for 30 min at 37°C followed by a 15-min wash in DHE-free Krebs. To quantitate the DHE fluorescence, the glass slides were placed under a Leica DM LB2 Fluorescent Microscope (Leica, Wetzlar, Germany) fitted with a rhodamine filter set (8).

### NO bioavailability assay

NO levels in the freshly isolated aortae were assessed using 4,5-diaminofluorescein diacetate (DAF-2 DA; Sigma-Aldrich, St Louis, MO), as described previously. Aortae were prepared in the manner described for the DHE assay and vessel sections were loaded with 5 μmol/l DAF-2 DA in the dark for 45 min. When the dye loading was complete, the vessels were rinsed three times with fresh Krebs solution. To quantitate the DAF-2 DA fluorescence, the glass slides were placed under the Leica DM LB2 fluorescent microscope fitted with a fluorescein isothiocyanate filter set (8).

### Statistical analysis

Data are the mean ± SEM. The statistical differences in mean values were assessed by the Student’s t-test. Two-sided p-values <0.05 were considered to indicate a statistically significant result.

## Results

### Establishment of hUCP2 transgenic mice

**Figure 1:**
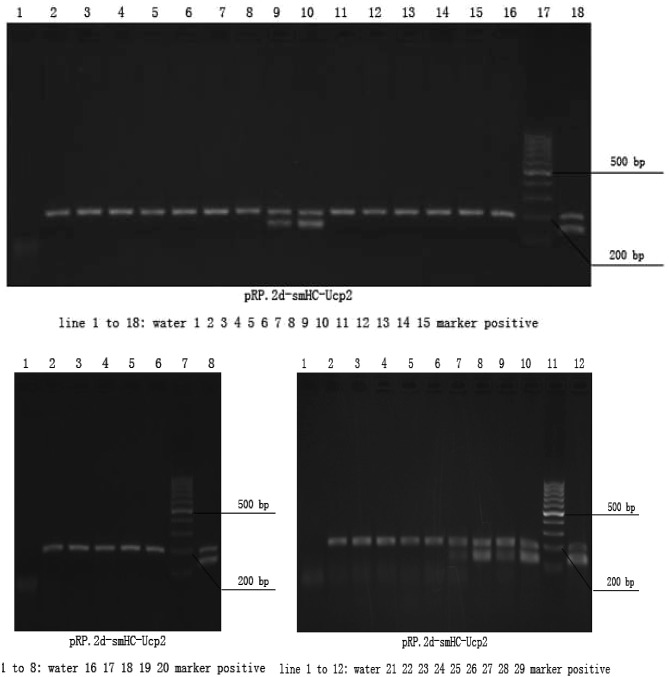
PCR results of transgenic mice. 8, 9, 26, 27, 28, 29: transgenic founder mice. Ucp2, uncoupling protein 2.

The transgenic fragments containing the full length rabbit smooth muscle myosin heavy chain promoter and hUCP2 cDNA were microinjected into the male pronuclei of 220 fertilized oocytes of FVB mice. A total of 160 injected eggs were implanted into the oviducts of 6 pseudo-pregnant foster mothers, which gave birth to 29 offspring. Six offspring mice were identified to be carrying the hUCP2 cDNA by PCR analysis (Fig. 1).

### UCP2 expression in aorta

**Figure 2:**
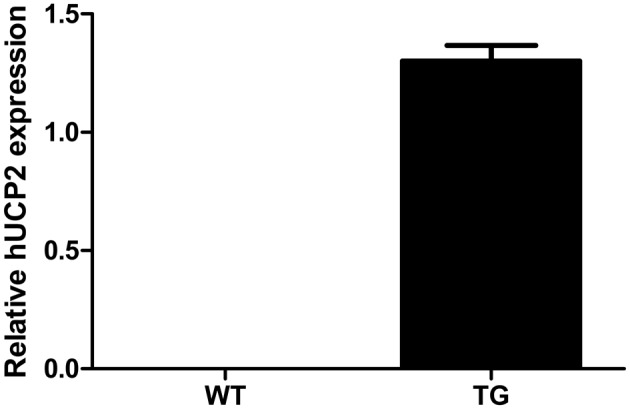
PCR results of hUCP2 expression in aorta. hUCP2, human uncoupling protein 2; WT, wild-type littermates; TG, transgenic mice.

Real-time PCR analysis confirmed that the transgenic construct was abundantly expressed in the aortae of the transgenic mice (TG) and that the UCP2 transcript was undetectable in the wild-type (WT) animals (Fig. 2).

### Superoxide level

**Figure 3:**
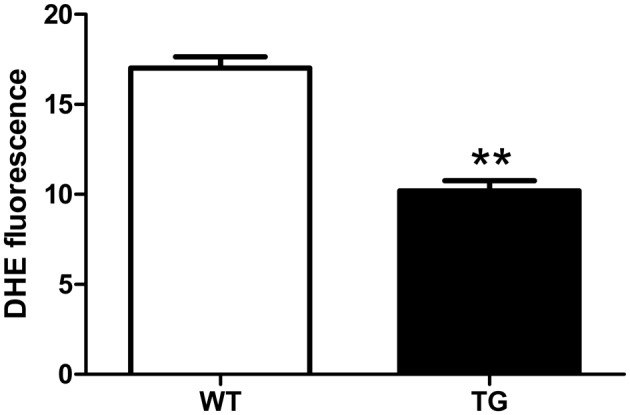
Superoxide production in aorta. Summarized data showing the average fluorescence intensity in aortae from each group. Values are the mean ± SEM; n=6 per group. WT, wild-type littermates; TG, transgenic mice. DHE, dihydroethidium. ^**^p<0.01 vs. the WT group.

Superoxide anion production in the aorta was assessed by DHE staining. We found that the DHE fluorescence was significantly lower in the aortae from the UCP2 TG mice than in those from their WT littermates (p<0.01; Fig. 3).

### NO availability

**Figure 4:**
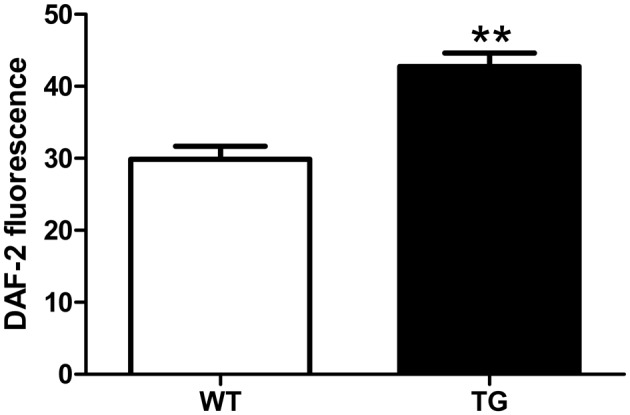
NO production in aorta. Summarized data showing the average fluorescence intensity in aortae from each group. Values are the mean ± SEM; n=6 per group. WT, wild-type littermates; TG, transgenic mice; DAF-2, 4,5-diaminofluorescein. ^**^p<0.01 vs. the WT group.

The production of NO in the aorta was assessed by DAF-2 DA staining. We found that DAF-2 DA fluorescence was significantly higher in the aortae from the UCP2 TG mice than in those from their WT littermates (p<0.01; Fig. 4).

## Discussion

In the present study, we report the establishment and initial characterization of a mouse model with the transgenic expression of human UCP2 in vascular SMCs under the control of the smooth muscle myosin heavy chain promoter. In the hUCP2 transgenic mouse, the hUCP2 mRNA was abundantly present in the aorta.

The aortae from the hUCP2 transgenic mice have significantly lower levels of superoxide and markedly higher levels of NO than the aortae from the WT mice. These characteristics may provide certain beneficial effects on the development of vascular dysfunction and remodeling.

In summary, we have established a transgenic mouse model with vascular SMC-specific overexpression of hUCP2. This model provides a novel tool for studies of the vascular effects of SMC-derived hUCP2.
